# Nano *Spirulina platensis* countered cisplatin-induced repro-toxicity by reversing the expression of altered steroid hormones and downregulation of the StAR gene

**DOI:** 10.1007/s00210-024-03483-z

**Published:** 2024-10-16

**Authors:** Eman M. Khalil, Mohamed I. Rady, Samah F. Darwish, Entsar R. Abd-Allah

**Affiliations:** 1 Department of Zoology, Faculty of Science (Girls), Al-Azhar University, Nasr City, Egypt; 2 Department of Zoology, Faculty of Science (Boys), Al-Azhar University, Nasr City, Egypt; 3 Biotechnology Research Unit, Animal Reproduction Research Institute, Giza, Egypt

**Keywords:** Cisplatin, Nano *Spirulina platensis*, StAR and SOD genes, DNA breakage, 17ß-HSD

## Abstract

Cisplatin is a commonly utilized chemotherapy medication for treating different sarcomas and carcinomas. Its ability interferes with cancer cells’ DNA repair pathways and postpones unfavorable outcomes in cancer patients. The current investigation’s goal was to ascertain if nano *Spirulina platensis* (NSP) might shield rat testicles from cisplatin damage by assessing the expression of the StAR and SOD genes, sex hormones, 17ß-hydroxysteroid dehydrogenase(17ß-HSD), sperm profile picture, oxidative condition of testes, testicular histology, and DNA damage. Four equal and random groups of 28 adult male Wistar rats were created; the control group was given saline for 8 weeks. An extraction of NSP at a concentration of 2500 mg/kg body weight was administered orally for 8 weeks to the NSP group. For the first 4 weeks, the cisplatin group was intraperitoneally injected with 2 mg/kg/body weight of cisplatin, and for the next 4 weeks, they were given a dosage of 4 mg/kg/body weight. The cisplatin + NSP group was given both NSP and cisplatin. The results of the experiment showed that intake of NSP and cisplatin improved sperm profile; re-established the balance of oxidizing agents and antioxidant state; enhanced testicular histology; promoted the histometric parameters of seminiferous tubules including epithelial height, their diameter, and Johnsen’s score, decreasing DNA breakage in testicular tissue; increased testosterone level; decreased 17ß-HSD concentration; and upregulated both the StAR and SOD gene expression in testicles compared to rats exposed to cisplatin alone. These results demonstrate that NSP is a promising agent for improving cisplatin-induced testicular injury and infertility.

## Introduction

Cis-[Pt-(NH_3_)_2_Cl_2_], also named CDDP or cisplatin, was the first transitional metallic complex to be applied as a chemotherapeutic drug and is one of the most used and influencing compounds in the treatment of cancer until now (Ghosh 2019). According to Dasari and Tchounwou (2014), it is among the most effective anticancer drugs against solid tumors in both adults and children, and the WHO has listed it as one of the “essential medicines” (WHO 2021). The year 2023 is expressed as the 45th year since the FDA agreement of cisplatin as a cancer drug, and, at present, it is broadly used alongside a range of human cancers, comprising neck and head, non-small cell lung cancer, primary-phase ovarian cancer, and progressive bladder cancer (Gandin et al. 2023). Also, testicular germ cell tumors can be treated by cisplatin (Keshta et al. 2023). It was found that the most excellent results are found versus testicular cancer, where cisplatin is 100% healthful if the cancer diagnosis is early. About 46% of patients receive a platinum-based drug in the clinics (Alassadi et al. 2022), and 10,000 papers every year have been published in the time 2020–2022, including cisplatin in the abstract, titles, or keywords (Gandin et al. 2023).

However, cisplatin disrupts normal cells, leading to toxicities in numerous organs, such as the testis and kidney (Choi et al. 2015; Liu et al. 2019). The cisplatin treatment caused germ cells, Sertoli cells, and Leydig cells at varying stages of maturity to be subjected to oxidative stress (Wang et al. 2020). Testicular tissues showed significant changes due to cisplatin, including increased expression of caspase 3 (Nofal et al. 2023). In addition, testicular fibrosis was caused by cisplatin injection, which also raised endoplasmic reticulum stress and mitochondrial damage as well as cellular apoptosis (Sharma and Sampath 2019; Wang et al. 2020). When people are healthy, their defense systems help to quench intracellular ROS and keep pro- and antioxidant levels. When ROS production exceeds normal antioxidant capacity, oxidative stress and the intrinsic cascade of apoptotic processes are triggered (Sharma and Sampath 2019; Chainy and Sahoo 2020). Male infertility, defined as reduced low sperm count, sperm motion, and heightened morphological abnormalities of sperm, has been linked to oxidative stress (Bansal and Bilaspuri 2010; Alahmar 2019; Aitken 2020). Consequently, it is necessary to sustain the anti-oxidation microenvironment and redox balance in the male genital organs to protect patients undergoing cisplatin-based chemotherapy treatments and maintain their fertility.

*Spirulina* is one of the most extensively applied natural components, representing a dietary complement for people, aquaculture, livestock, and poultry, and having proteins, fatty acids, chlorophyll, vitamins, minerals, and necessary amino acids as well as significant amounts of rare natural antioxidants such phycocyanin, carotenoids, and polyphenols (El-Shall et al. 2023). Excellent value proteins, 18 of the 20 specific amino acids, calcium, potassium, all B vitamins, vitamins A, E, and K, besides numerous vital enzymes and minerals, are all included in *Spirulina* powder (Ebid et al. 2022). Immunomodulation, antioxidant, anticancer, antiviral, and antibacterial properties, in addition to protective influence alongside anemia, radiation damage, hyperlipidemia, diabetic disease, obesity, stimulating hypersensitive reactions, toxicity caused by heavy metals or chemicals, and malnutrition, are some of the potential health benefits of consuming *Spirulina* (Wu et al. 2016; El-Shall et al. 2023). Furthermore, in people who received *Spirulina* complements, the pro-inflammatory reduced significantly and the concentrations of IFN-g, which has antiviral action and significant immunoregulatory roles, pointedly improved (Aghasadeghi et al. 2024).

Human serum is rich in dehydroepiandrosterone (DHEA), a precursor of sex hormones such as testosterone. Its concentration is higher between the ages of twenty and thirty and gradually decreases with aging, elevating many pathological diseases in older adults (Vegliante and Ciriolo 2018).

In endocrine organs, including testes, the intracellular transfer of cholesterol to the mitochondrion is crucial for producing steroidal hormones dependent on the steroidogenic acute regulatory (StAR) protein (Burget et al. 2018). StAR is greatly produced by Leydig cells, and it interacts with a protein complex at the outer membrane of mitochondria including the translocator protein (Galano et al. 2021).

Oxidative stress is one of the important reasons associated with male infertility (Cito et al. 2020). Therefore, any genetic polymorphism occurring in one of the antioxidant genes may increase the threat of male infertility. SOD1 and SOD2 are key in removing ROS and keeping cells from oxidative stress and free radicals (Bach et al. 2023). It was found that SOD1 deficiency impaired fertility of mouse sperm (Tsunoda et al. 2012) or reduced spermatogonia in sod1-knockout mice initiated by a high degree of temperature (Ishii et al. 2005). Bach et al. (2023) observed that 7958GA of SOD1 in the sterile men was notably more significant than that in control men indicating that the GA genotype may threaten human male infertility. Earlier studies have demonstrated that the SOD2 c.47 T > C(rs4880) polymorphism was correlated to male infertility (Karam et al. 2022).

No previous work examined whether NSP is a shielding agent against cisplatin-related infertility. Because of its advantages for bioavailability and longevity due to its nano-configuration, nano *Spirulina platensis* (NSP) was employed in this experiment. It is predicted that NSP will be exploited as a very successful antioxidant agent. This work aimed to confirm if NSP may keep male rats receiving cisplatin in good reproductive health.

This experiment was directed through biochemical analysis of the reproductive hormones, the steroidogenic enzymes, the oxidative stress markers, the morphometric measurements of both diameter and epithelial germinal layer height of seminiferous tubule, as well as Johnsen’s score estimation, DNA damage analysis of testicular tissue, the sperm profile characteristics, and finally expression of StAR and SOD genes in the testicles.

## Materials and methods

### *Spirulina platensis* (SP) yield and preparation of extraction

Fresh SP leaves were obtained from the National Research Centre in Dokki, Giza, Egypt. The leaves were dried before processing via an electric mixer to make powder. To get the extraction, 250 g of powder was steeped in 2.5 L of methanol for 6 h at 70 °C. Then, 10% methanol (W/V) concentration was used to mix with this powder extraction (Laughton et al. 2021).

### Nano *Spirulina platensis* (NSP) synthesis

The ball mill process is a popular and effective way to produce *Spirulina*, a cyanobacterium recognized for its high protein content and nutritional benefits. This procedure involves crushing the raw ingredients in a ball mill to produce a fine powder that can then be used to cultivate *Spirulina* (Yadav et al. 2012). Here is an outline of the synthesis process with the ball mill method:

1. Raw material selection: *Spirulina* synthesis typically requires a carbon source (sodium bicarbonate) and a nitrogen source (sodium nitrate). Trace elements and minerals such as potassium, magnesium, and calcium may also promote growth.
2. Raw material preparation: Appropriately mix the selected raw materials to form a homogenous mixture. This mixture will act as a growth medium for *Spirulina*.
3. Grinding with a ball mill: Place the prepared raw material mixture in the ball mill. The ball mill is a cylindrical container packed with balls of various sizes made of stainless steel, ceramic, or zirconia. The container is then shut and rotated at a predetermined pace, forcing the balls to collide with one another and the raw material combination. This technique finely grinds the combination, increasing surface area and nutrient bioavailability for *Spirulina* growth.
4. Sieving and washing: After grinding, strain the powdered mixture through a sieve to remove any larger particles. Next, thoroughly wash the powder to remove impurities, such as unground raw materials or pollutants.
5. pH adjustment and inoculation: Set the pH of the cleaned powder to the appropriate level for *Spirulina* development (usually around pH 10). Inoculate the powder with a small amount of *Spirulina* culture to commence the growing process.
6. Cultivation: Place the infected powder in photobioreactors or open ponds with the appropriate light, temperature, and aeration conditions for *Spirulina* development. The *Spirulina* culture will develop and multiply over time, producing biomass that may be harvested and processed for various uses, including food, animal feed, and nutraceuticals (Soni et al. 2017).

### Characterization of nano *Spirulina platensis* (TEM)

A transmission electron microscope (TEM, JEOL JEM-1400) assessed the NSP geomorphology. To conduct the analysis, a droplet of the dilute sample was put into a copper grid and dyed with 2% (w/v) phosphor tungstic acid. Amplification of 40,000 × was adjusted to take pictures at 120 kV (Pyrz and Buttrey 2008).

### Limit test: NSP safety

The LD_50_ cut-off value of NSP was determined using the limit test. With the Microsoft Excel tool, eight rats weighing 165–185 g (8 weeks old) were randomly assigned to dose classes based on the weight of the animals.

### Experimental animals

The National Organization for Drug Control and Research in Giza, Egypt, supplied 28 adult Wistar rats (*Rattus norvegicus*) (8 weeks old) weighing 165–185 g. The animals were placed in collections of four or five plastic boxes. Ecological conditions for rats’ accommodation were adjusted at a 12-h light/12-h dark sequence and 20–25 °C, 55–65% moisture. The rats were given their usual pellet food and water while remaining in an environment free of microbes. Cairo University Ethics Committee (CU/IF 3/22) examined and approved the animal population, study design, and animal treatment. A detection study began with a 5000 mg/kg dose for the first rat. Subsequently, four more animals were dosed at this level. A period of at minimum 24 h will be permitted between the dosing of each rat. All animals should be monitored for at least 14 days. The other three rats remain as a control for outcomes comparison. The body weight of animals was measured on experimental days 0, 7, and 14. The animals were observed for toxicity signs (such as tears, hair loss, body weight gain and loss, yellow hair, and loss of appetite). Each group had all its dead animals documented.

### Research categories

We used four groups of rats (7 rats each). Along with Creasy (1997), the experiment’s 8 weeks extent corresponds to the termination of spermatogenesis. These sets were along these lines.

- Control group: animals received saline by gavage.
- NSP group: rats were orally administered NSP at 2500 mg/kg bw (half of LD_50_).
- Cisplatin group: Rats were intraperitoneally injected with cisplatin at a dosage of 2 mg/kg/body weight for the first 4 weeks (González et al. 2023), followed by a dosage of 4 mg/kg/body weight for the second 4 weeks (Seto et al. 2016).
- Cisplatin + NSP group: animals were given both NSP and cisplatin.

Following the final dosage, all rats were intraperitoneally injected with sodium pentobarbital (60–100 mg/kg bw) for anesthetization. Blood samples were collected from each drugged animal via a cardiac puncture and placed in a test tube for serum preparation. The animals’ epididymis, seminal vesicles, and testes were removed upon dissection. These organs were washed using normal saline, dried in air, and weighed.

### Hormone investigation

The concentrations of dehydroepiandrosterone (DHEA) and testosterone (TS) in blood sera were determined using the Sun Long Biotech Co., LTD, China (Mainland), method, using a competitive enzyme immunoassay technique (ELISA kits).

### Testicular enzyme activities

17-Beta-hydroxysteroid dehydrogenase(17ß-HSD) activity is measured via ELISA kits (Technical Manual, Catalog# RTEB1201).

### Epididymal sperm concentration

The cauda epididymal tissues were removed and immersed in a petri dish containing 1 mL of physiological saline (pH = 5.5). All parts were quickly macerated with razor-sharp scissors before releasing their sperms into the physiological saline. The Neubauer chamber and a microscope were used to determine the total number of spermatozoa (Saalu et al. 2007). The sperm count was estimated by calculating the total number of sperms in 5 squares in RBC as follows:

Total number of sperms = (Total number of sperms per square (×)/Total volume per square (10)^−4^) × dilution factor (20)

### Sperm motility

Sperm solution suspension was placed on a clean, formerly heated slide covered with a covering slip. To determine spermatozoa movement, an optical microscope containing a 40 × objective (Morrissey et al. 1988) was used.

### Assessment of the viability and morphology of sperm

Sperm solution suspension (10 µL) was placed on a slide and dyed with eosin and nigrosine staining. Once sperms die, the cell membrane frequently ruptures, allowing the stain to penetrate the cytoplasm of the spermatozoon (Morakinyo et al. 2009). Total abnormalities, such as a shapeless head, large or small head, or tail shape, were recorded.

### Fructose level determination in the seminal vesicle

Excessive fructose concentrations in sperm are widespread, and this sugar is obtained from seminal vesicles. The fructose quantity in human sperm and the fructolysis indicators were assessed to inspect if there was a relation between two glycolysable sugars and sperm count. The resorcinol technique was used to determine the fructose level in the plasma of seminal vesicles. Fructose acts with resorcinol in a high concentration of hydrochloric acid solution to produce a crimson chemical. Using a wavelength of 560 nm, the color pattern of fructose and zinc to blanks can be compared (Foreman et al. 1973).

### Determining the oxidative status in rat testicles

To make the tissue homogenate, each rat’s right testicle was removed and standardized in PBS (1:10 mL). This mixture was centrifugated at 4000 rpm for 20 min, and the supernatant residue was easy to measure malondialdehyde (MDA) (Satoh 1978), glutathione (GSH) (Beutler 1963), and superoxide dismutase (SOD) and catalase (CAT) (Weydert and Cullen 2010).

### Histological and morphometric examination

A neutral formalin solution (10%) was used to fix the testes samples. After 24 h, the tissue was cleaned for 24 h with tap water, dehydrated with declining concentrations of alcohol, cleaned in xylene, and then embedded in paraffin wax. The slices (4–5 µm thick) were stained by hematoxylin and eosin (Suvarna et al. 2018). The slides were checked by an Olympus BX41 light microscope, provided with a digital camera, and the image analysis computer program ImageJ software (NIH, Bethesda, MD, USA) was used to analyze the captured photos (Rasband 2014). Around ten selected fields of more circular or just about spherical seminiferous tubule sections were chosen from each group, and their average mean was determined (Yonezawa et al. 2018). Germinal epithelium height and tubular diameter were captured via the histological slides stained by H&E (× 10). A previously established technique (Johnsen 1970) was used by Antonuccio et al. (2020) to calculate the mean Johnsen’s testicular biopsy score (MJTBS), as shown in Table 1.

**Table 1:** Mean Johnsen’s testicular biopsy score (MJTBS)

Description	Score
Tubular sclerosis, no seminiferous epithelial cells	1
Only Sertoli cells, no germ cells	2
Only spermatogonia	3
No spermatids, arrest of spermatogenesis at the primary spermatocyte stage	4
Many spermatocytes but no spermatids	5
No late spermatids, arrest of spermatogenesis at the spermatid stage	6
Many early spermatids but no late spermatids	7
Few late spermatids	8
Disorganized tubular epithelium with several late spermatids	9
Full spermatogenesis	10

### Analysis of DNA damage in testicular tissue by comet assay

Single-cell gel electrophoresis and alkaline comet investigations were carried out according to Araldi et al. (2015). Testicular tissue samples from rats were mixed with 10% tissue solution in cooled homogenate buffer (pH 7.5) consisting of 24 mM Na2EDTA (pH 13) and 75 mM NaCl for successful homogenization. A fluorescence microscope with 400 magnification (Carl Zeiss Axioplan with epifluorescence employing filters 15 BP546/1 2, FT580, and LP590) and a closed-circuit digital camera were employed for slide investigation. Using the COMET five image analysis instrument, 50 cells were acquired for each sample.

### Study of StAR and SOD gene expression

#### Total RNA extract and cDNA formation

Total RNA was isolated from testis tissue by TRIzol total RNA extract reagent using the TRIzol kit methods (catalog number 15596–026 from Life Technologies). RNA was subsequently reverse-transcribed into complementary DNA (cDNA) strands by the Sensi Fast cDNA synthesis kit (catalog number BIO-65053).

#### Quantitative real-time reverse transcription PCR (qRT-PCR) analysis

The mRNA concentration of StAR and SOD genes was determined using a quantitative RT-SYBR green PCR technique. The GAPDH gene acted as a reference gene for mRNA quantification. The primers used for amplification of three genes are provided in Table 2.

**Table 2:** GAPDH, StAR, and SOD Wistar rat cDNA primer sequences

Gene	Sense 5′–3′	Antisense 5′–3′	Product size (bp)
GAPDH	AGACAGCCGCATCTTCTTGT	TGATGGCAACAATGTCCACT	142
StAR	CCTGAGCAAAGCGG TGTCAT	GCAAGTGGCTGG CGAACTCTA	187
SOD	GCAGAAGGCAAGCGGTGAAC	TAGCAGGACAGCAGATGAGT	387

Maxima SYBR qPCR master mix (Thermo Scientific) and the Rotor-Gene 6000 real-time PCR equipment (Qiagen, Germany) were used for the reaction and detection of qRT-PCR, respectively (Rucinski et al. 2008; Coccini et al. 2014).

The PCR cycling settings involved a primary activation step at 95 °C for 10 min, after that, 40 cycles of 10 s at 95 °C, 15 s at 60 °C for the StAR gene and 58 °C for the SOD gene, and 20 s at 72 °C. After that, the melt curve was analyzed.

The reaction volume was 15 µL and contained 1 × Maxima SYBR Green qPCR master mix, 7.5 pmol of each primer, and 0.75 µL of cDNA.

The comparative cycle threshold approach (2 -ΔΔCT) was used to determine the DNA level and relative fold change in expression.

### Statistics

The mathematical data were estimated using GraphPad Prism 8 software and stated as mean ± SE, then compared between the different groups by one-way ANOVA (one-way analysis of variance) followed by Tukey HSD multiple range tests. The meaningful degree was expressed at 5% (*P* < 0.05).

## Results

### Result of acute toxicity

Nano *Spirulina* experienced a half-lethal dose of over 5000 mg/kg/BW. There were no signs of toxicity or mortality. Also, there was no significant difference in body weight changes between the control and treated groups (Table 3).

**Table 3:** Evaluation of LD_50_ of nano *Spirulina platensis*

Responses to dose	Doses	
	Control mg/kg	5000 mg/kg
Number of animals	3	5
Number of live animals	3	3
Number of dead animals	0	0
Signs of toxicity	No	No
Body weight of animals at the day of dosing	174.5	189.5
Body weight of animals after the first week	186	195.3
Body weight of animals after the second week	194	**212**
Body weight change	19.5	**22.5**

### Result of TEM

**Fig. 1:**
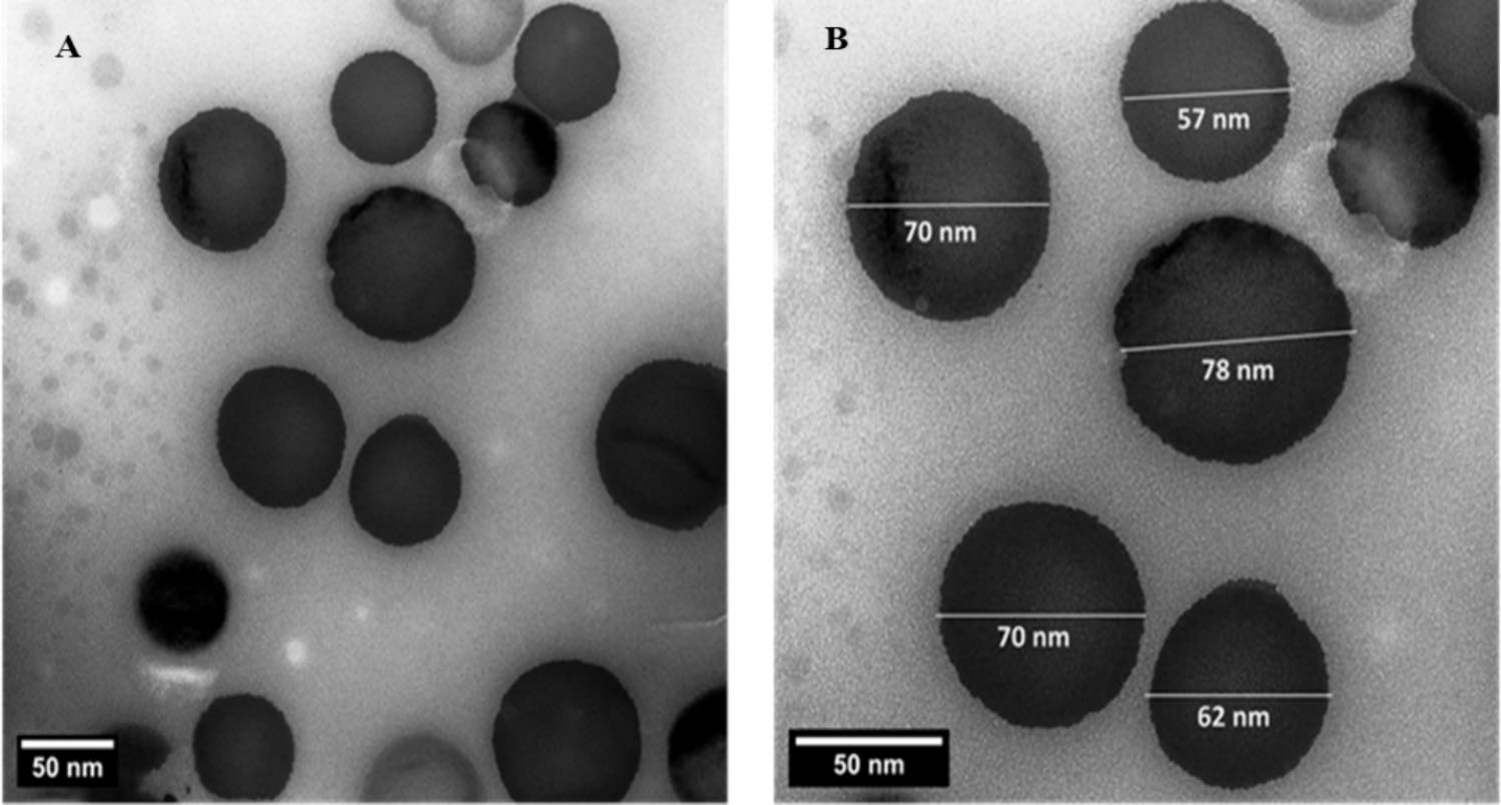
HR-TEM image of NSP shows spherical shape particles with average diameter around 68 nm

Transmission electron microscope images of NSP nanoparticles display their sphere-shaped nature. These nanoparticles were about 68 nm in diameter (Fig. 1).

### Impact of NSP on body mass gain and the weight of sexual organs

**Fig. 2:**
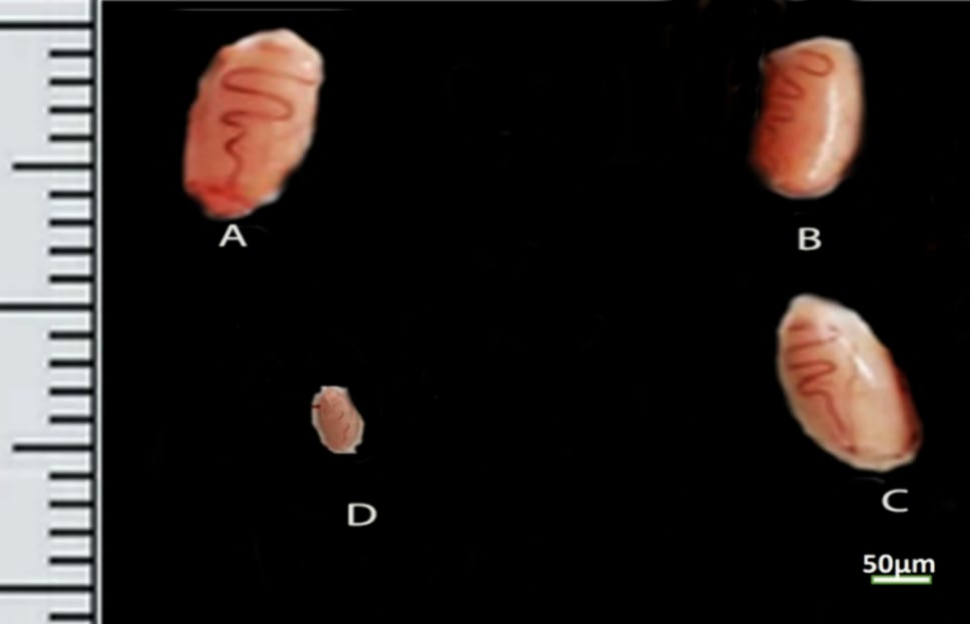
Photomicrographs of the rat testes of four experimental groups at day 56: **A** control group, **B** NSP group, **C** NSP + cisplatin group, **D** cisplatin group

The gain in body mass was expressively less in the cisplatin-treated rats (27.5 ± 5.93) when compared to the control (100.5 ± 6.14), NSP (87.83 ± 5.78), and cisplatin + NSP (36.83 ± 3.36) groups. The seminal vehicles’ absolute weight in both controls (1.59 ± 0.06) was pointedly boosted compared to the cisplatin group (0.96 ± 0.26). The left and right cauda epididymis absolute weight in the cisplatin-treated rats (0.22 ± 0.02) (0.15 ± 0.04), respectively, was notably decreased when compared to the control rats (0.33 ± 0.02) (0.32 ± 0.01), respectively. Both left and right testicles’ absolute weights (1.00 ± 0.14) (0.89 ± 0.20), respectively, were pointedly diminished in the cisplatin-treated rats when compared to the control animals (1.75 ± 0.05) (1.64 ± 0.07), respectively (Tables 4 and 5; Fig. 2).

**Table 4:** The body weight gain (g) and absolute reproductive organ weight (g)

Parameters	Groups			
	Control	NSP	Cisplatin	Cisplatin + NSP
Body weight gain	100.50 ± 6.14	87.83 ± 5.78	27.50 ± 5.93^*$#^	36.83 ± 3.36^*$^
Right testes	1.64 ± 0.07	1.60 ± 0.08	0.89 ± 0.20^*$^	1.30 ± 0.15
Left testes	1.75 ± 0.05	1.78 ± 0.07	1.00 ± 0.14^*$^	1.27 ± 0.13^*$^
Right cauda epididymis	0.32 ± 0.01	0.31 ± 0.02	0.15 ± 0.04^*$^	0.27 ± 0.02
Left cauda epididymis	0.33 ± 0.02	0.28 ± 0.01	0.22 ± 0.02^*^	0.25 ± 0.02
Seminal vesicle	1.59 ± 0.06	1.51 ± 0.11	0.96 ± 0.26^*#^	1.80 ± 0.08

Results are expressed as mean ± standard error of mean. The superscripts *, $, and # display the significant difference (*P* value < 0.05) between groups in comparison to the control, NSP, and cisplatin + NSP groups, respectively

**Table 5:** The relative reproductive organ weight (%)

Parameters	Group			
	Control	NSP	Cisplatin	Cisplatin + NSP
Right testes	0.58 ± 0.04	0.60 ± 0.03	0.52 ± 0.11	0.58 ± 0.06
Left testes	0.62 ± 0.02	0.67 ± 0.02	0.59 ± 0.08	0.57 ± 0.06
Right cauda epididymis	0.12 ± 0.01	0.10 ± 0.02	0.09 ± 0.02	0.12 ± 0.01
Left cauda epididymis	0.12 ± 0.01	0.10 ± 0.01	0.13 ± 0.01	0.12 ± 0.01
Seminal vesicle	0.56 ± 0.03	0.58 ± 0.04	0.56 ± 0.15	0.82 ± 0.04

Results are expressed as mean ± standard error of mean. The superscripts *, $, and # display the significant difference (*P* value < 0.05) between groups in comparison to the control, NSP, and cisplatin + NSP groups, respectively

### Sperm profile outcomes

**Fig. 3:**
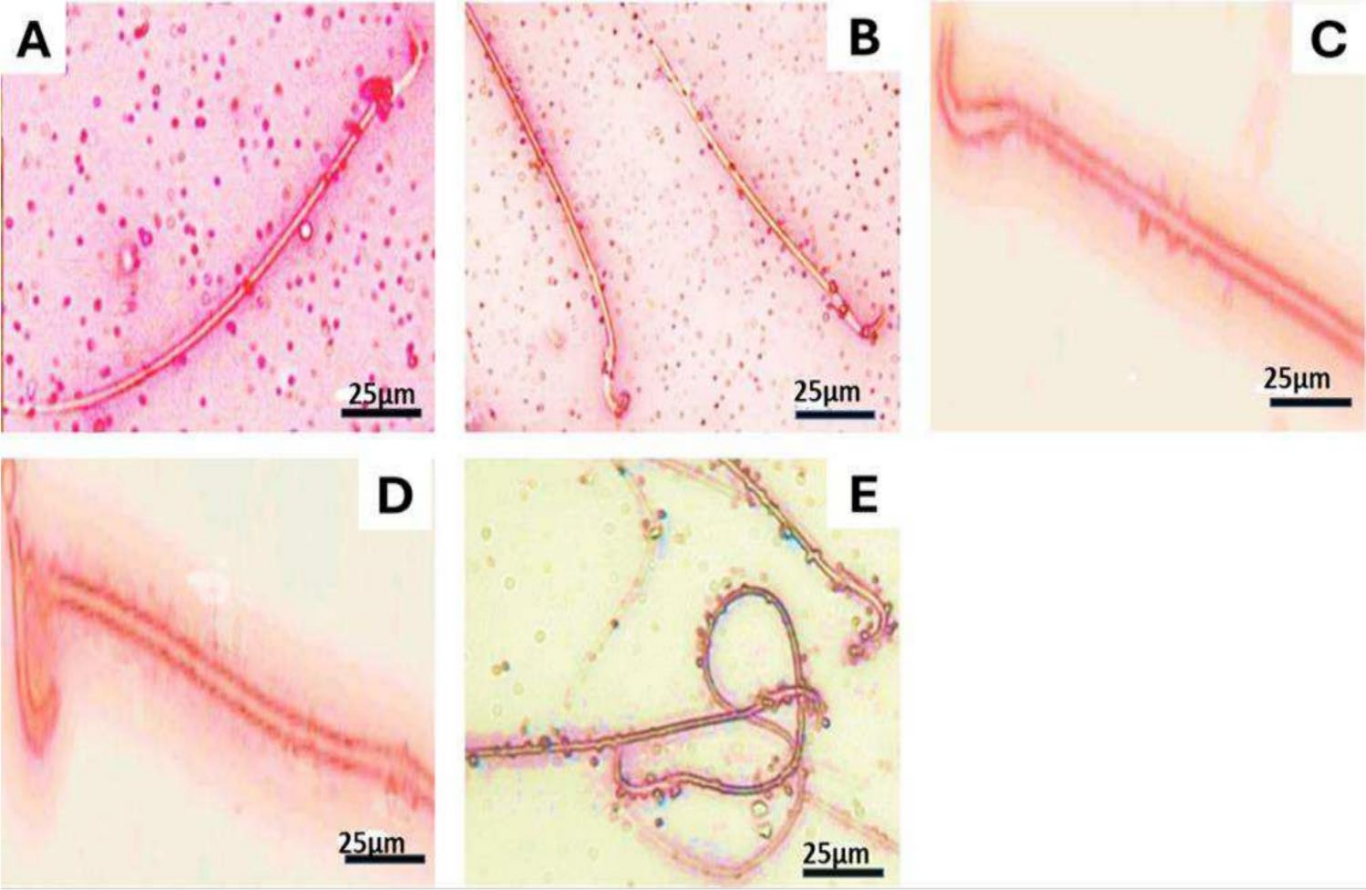
Photomicrographs showing sperm morphological abnormalities. **A** normal live sperm. **B** On the right, normal live sperm; on the left, banana shape. **C** Hookless head. **D** Amorphous head. **E** Abnormal tail

Cisplatin treatment showed oligospermia. In addition, the cisplatin-treated rats presented a significant rise in fructose level (318.33 ± 3.84) compared to the control group (178.66 ± 8.11). The cisplatin-treated group showed many sperm abnormalities, including hookless, amorphous head, and abnormal tail (Tables 6 and 7; Fig. 3).

**Table 6:** Fructose level, sperm count, motility, and viability (%) after 8 weeks

Parameters	Groups			
	Control	NSP	Cisplatin	Cisplatin + NSP
Fructose level	178.66 ± 8.11	231.00 ± 14.73^*^	318.33 ± 3.84^*$#^	271.33 ± 5.78^*^
Count × 10^6^/mL	127.33 ± 1.45	123.33 ± 4.40	Oligospermia	89.33 ± 2.33^*$^
Viability (%)	72.33 ± 1.45	77.66 ± 1.45	Oligospermia	63.00 ± 1.52^*$^
Motility (%)	56.33 ± 0.88	52.66 ± 1.45	Oligospermia	42.66 ± 1.85^*$^

Results are expressed as mean ± standard error of mean. The superscripts *, $, and # display the significant difference (*P* value < 0.05) between groups in comparison to the control, NSP, and cisplatin + NSP groups, respectively

**Table 7:** Sperm morphological abnormalities in different groups

Parameter	Groups			
	Control	NSP	Cisplatin	Cisplatin + NSP
Total abnormality sperm, %	17.00 ± 0.57	15.33 ± 1.45	Oligospermia	24.66 ± 0.88^*$^
Hookless, %	1.33 ± 0.33	0.33 ± 0.33	Oligospermia	4.00 ± 0.57^*$^
Amorphous head, %	6.66 ± 0.33	4.00 ± 0.57^*^	Oligospermia	15.00 ± 0.57^*$^
Banana head, %	1.66 ± 0.33	0.66 ± 0.33	Oligospermia	2.66 ± 0.88
Abnormal tail, %	12.66 ± 0.33	14.00 ± 0.57	Oligospermia	21.66 ± 0.88^*$^

Results are expressed as mean ± standard error of mean. The superscripts *, $, and # display the significant difference (*P* value < 0.05) between groups in comparison to the control, NSP, and cisplatin + NSP groups, respectively

### Reproductive hormones

The cisplatin-treated group presented a noticeable decline in DHEA and TS (304 ± 5.68) (5.23 ± 0.18) when compared to the untreated animals (421.7 ± 2.18) (6.36 ± 0.14), respectively. The combined group showed a substantial increase in DHEA and TS (398 ± 1.52) (6.16 ± 0.12) when compared with the cisplatin-treated group (304 ± 5.68) (5.23 ± 0.18), respectively (Table 8). This means that the NSP group restored the concentration of these sex hormones.

**Table 8:** The male reproductive hormone levels in different groups

Parameter	Groups			
	Control	NSP	Cisplatin	Cisplatin + NSP
TS (ng/mL)	6.36 ± 0.14	6.23 ± 0.26	5.23 ± 0.18^*$#^	6.16 ± 0.12
DHEA (ng/dL)	421.7 ± 2.18	430 ± 5.77	304 ± 5.68^*$#^	398 ± 1.52^*$^

Results are expressed as mean ± standard error of mean. The superscripts *, $, and # display the significant difference (*P* value < 0.05) between groups in comparison to the control, NSP, and cisplatin + NSP groups, respectively

### Effect of cisplatin on 17ß-HSD13

**Fig. 4:**
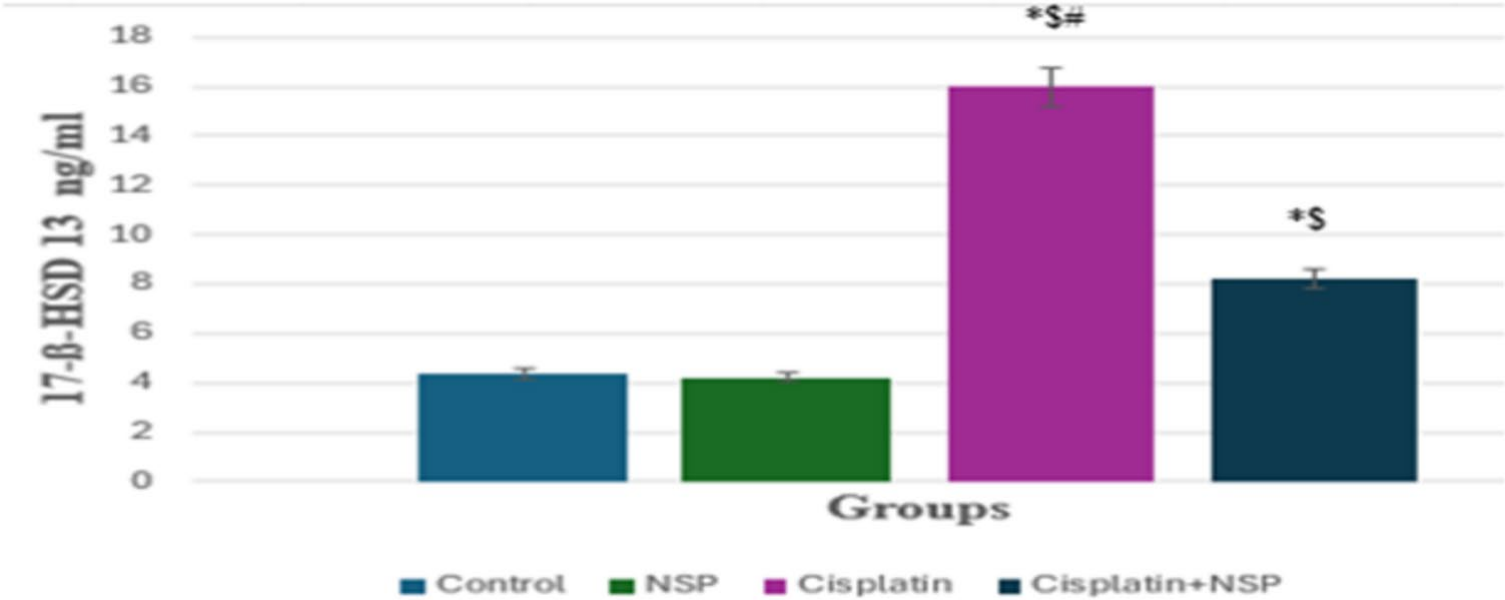
Effect of cisplatin and NSP on the concentration of 17-ß-HSD 13. Bars are displayed based on mean and SEM values. * means statistically significant as compared to control; $ means statistically significant as compared to the NSP group; # means statistically significant as compared to the cisplatin + NSP group at *p* < 0.05

The result showed that treatment with cisplatin significantly increased the concentration of 17-ß-HSD 13 (15.96 ± 1.01) compared to the control group (4.40 ± 0.21). On the other hand, NSP improved the level of 17-ß-HSD 13 (8.20 ± 0.47) (Fig. 4).

### Oxidative status

Glutathione (GSH), catalase (CAT), and superoxide dismutase (SOD) concentrations significantly declined after cisplatin injection as compared to the control group. Malondialdehyde (MDA), a lipid peroxidation marker, intensified noticeably after cisplatin administration compared to untreated rats. Nano SP treatment minimized the ROS level that was increased by cisplatin treatment. In comparison to the cisplatin-treated rats, NSP was capable of distinctly improving CAT, GSH, and SOD activity as well as decreasing the level of MDA (Table 9).

**Table 9:** The level of testicular oxidative stress markers in different groups

Parameters	Groups			
	Control	NSP	Cisplatin	Cisplatin + NSP
CAT (U/mg protein)	81.25 ± 2.68	96.00 ± 1.87^*^	52.75 ± 2.01^*$#^	78.00 ± 3.39^$^
GSH (U/g tissue)	3.08 ± 0.11	3.33 ± 0.29	1.97 ± 0.11^*$#^	3.11 ± 0.18
SOD (U/g tissue)	941.25 ± 3.72	901.50 ± 3.92^*^	585.00 ± 6.33^*$#^	814.75 ± 4.38^*$^
MDA (nmol/g tissue)	85.50 ± 4.62	90.50 ± 3.66	188.50 ± 3.37^*$#^	145.00 ± 4.02^*$^

Results are expressed as mean ± standard error of mean. The superscripts *, $, and # display the significant difference (*P* value < 0.05) between groups in comparison to the control, NSP, and cisplatin + NSP groups, respectively

### Histopathological observations

**Fig. 5:**
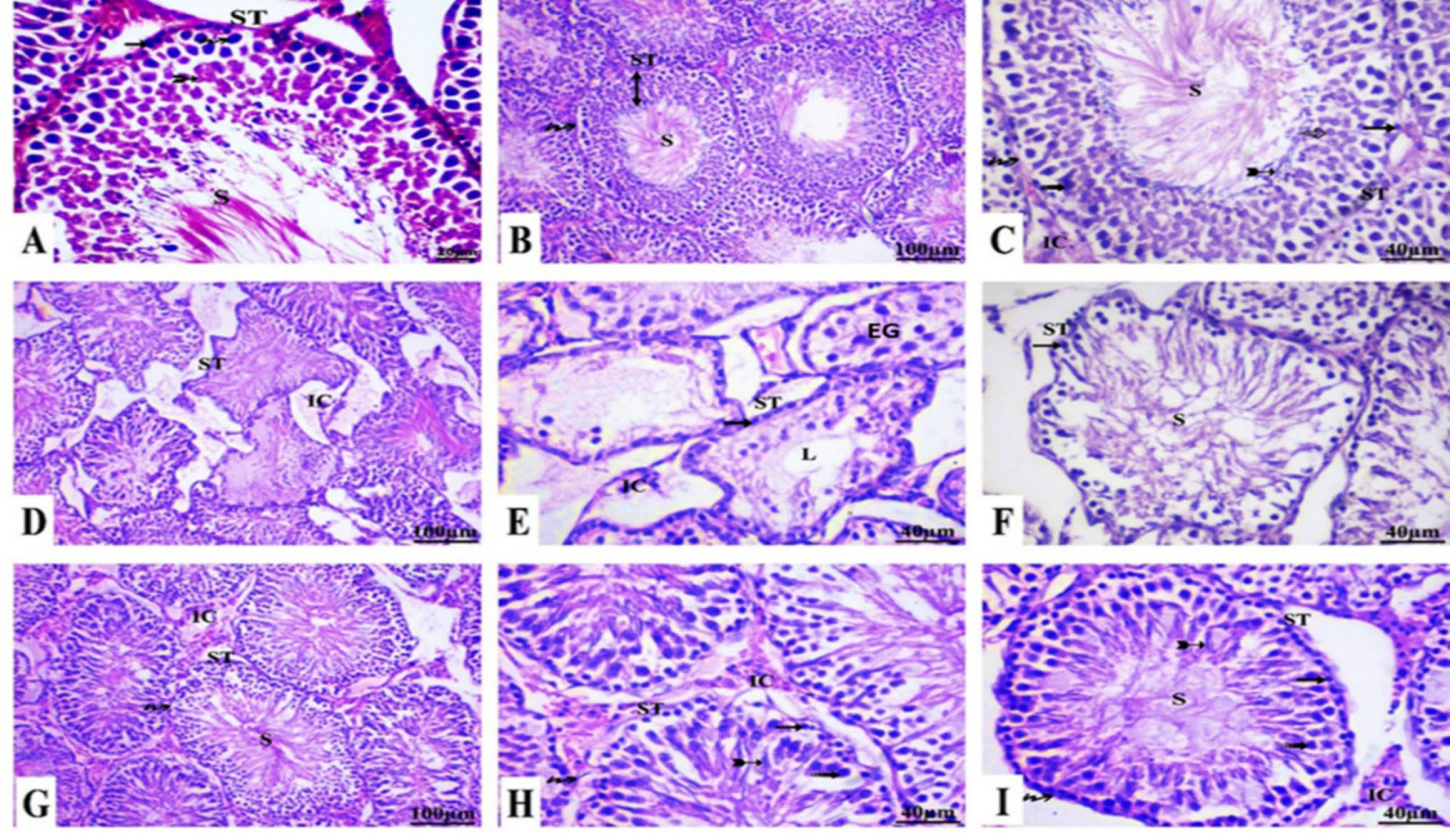
Histopathological H&E sections of testis among different groups. **A** Transverse section from the testis of the control group showing normal spermatogonia (arrow), primordial spermatocytes (wavy arrow), round spermatids (bifid arrow), spermatozoa (S), and interstitial cells (asterisk) situated amidst the seminiferous tubules (ST). **B**, **C** Transverse sections from the testis of NSP-treated group showing spermatogonia (arrow), basement membrane (wavy arrow), primordial spermatocytes, round spermatids (hollow arrow), spermatozoa (S), and interstitial cells (IC). **D**–**F** Transverse sections from the testis of the cisplatin-treated group showing pronounced destruction of seminiferous tubules (ST), vanishing of spermatocyte and spermatid as well as absence of identifiable sperm, solely spermatogonia (arrow), exfoliated germ cells in the lumen (EG), and a decrease in interstitial cells (IC). The majority of the observed ST exhibited irregular shapes without distinct forms, accompanied by significant disruption in the pattern of spermatogenic cells. Disappearance of spermatocyte and spermatid as well as absence of identifiable sperm within the lumen. Most cells exhibited degeneration. A considerable number of spermatogenic cells demonstrate areas of reduction. The germinal epithelium displayed prominent intercellular gaps and, in certain cases, areas of localized loss. **G**–**I** Transverse sections from the testis of the combined group (NSP + cisplatin) showing predominantly normal histological structures; seminiferous tubules (ST), well-defined basement membranes (wavy arrow), spermatogonia (arrow), primary spermatocytes (dotted arrow), round spermatids (hollow arrow), elongated spermatids (bifid arrow), spermatozoa (S), and a population of Leydig cells or interstitial cells (IC)

A progressive arrangement of several types of spermatogenic cells of the testes in both control and NSP groups (Fig. 5A–C) was perceptive. Spermatogonia were observed at the basement membrane, followed by primordial spermatocytes and spherical spermatids, and the lumen contained spermatozoa. Interstitial areas with Leydig cells between the seminiferous tubules were noticed. The testis of the cisplatin group (Fig. 5D–F) showed abnormal spermatogenesis and a marked shrinkage of seminiferous tubules with wide lumen. Also, most of the identified seminiferous tubules had irregular shapes, a considerable imbalance in the arrangement of spermatogenic cells, exfoliation of spermatocytes and spermatids, and a lake of sperms inside the lumen. In addition, a significant decrease in spermatogenic and interstitial Leydig cells was noticed, and the germinal epithelium showed significant intercellular gaps (Fig. 5E). The cisplatin + NSP exhibited an apparent protective effect of NSP against cisplatin adverse effects; the seminiferous tubules generally have regular histological features like those of the control group. The tubules exhibited a well-defined basement membrane, normal spermatogonia, primary spermatocytes, spermatids, and sperms, in addition to Leydig cells in the interstitial spaces (Fig. 5G–I).

### Histometric measurements

#### Germinal epithelial height and diameter of seminiferous tubules

Cisplatin administration triggered a crucial reduction in seminiferous tubule diameter and germinal epithelial height (132.31 ± 7.20 µm) (10.6 ± 0.62 µm) when compared with the control group (176.1 ± 4.60 µm) (41.1 ± 1.80 µm), respectively. However, rats treated with both cisplatin and NSP improved these parameters (167.9 ± 8.40) and (37.1 ± 3.7), respectively, when compared to the cisplatin group (Table 10; Fig. 5).

**Table 10:** Histometric analysis of seminiferous tubule in different groups

Parameters	Groups			
	Control	NSP	Cisplatin	Cisplatin + NSP
Seminiferous tubule diameter (µm)	176.1 ± 4.60	181.8 ± 8.50	132.31 ± 7.20^*$#^	167.9 ± 8.40
Seminiferous tubule epithelium height (µm)	41.1 ± 1.80	46.6 ± 3.86	10.6 ± 0.62^*$#^	37.1 ± 3.7
Johnsen testicular score	9.9 ± 0.06	9.8 ± 0.12	4.2 ± 0.60^*$#^	8.4 ± 0.20^*$^

Statistical data were estimated using GraphPad Prism 9 software and expressed as the mean ± standard error of mean; number of replicates is *n* = 10. The superscripts *, $, and # display the significant difference (*P* value < 0.05) between groups in comparison to the control, NSP, and cisplatin + NSP groups, respectively

#### Johnsen testicular tissue score

Outcomes in Table 10 estimate the histological development of germ cells, spermatogenesis, and spermatocytogenesis. There was a substantial difference between the control (9.9 ± 0.06) and the cisplatin group (4.2 ± 0.60). Conversely, the NSP group restored the development of spermatogenesis (8.4 ± 0.20).

#### Cisplatin genotoxicity assessment by comet assay

**Fig. 6:**
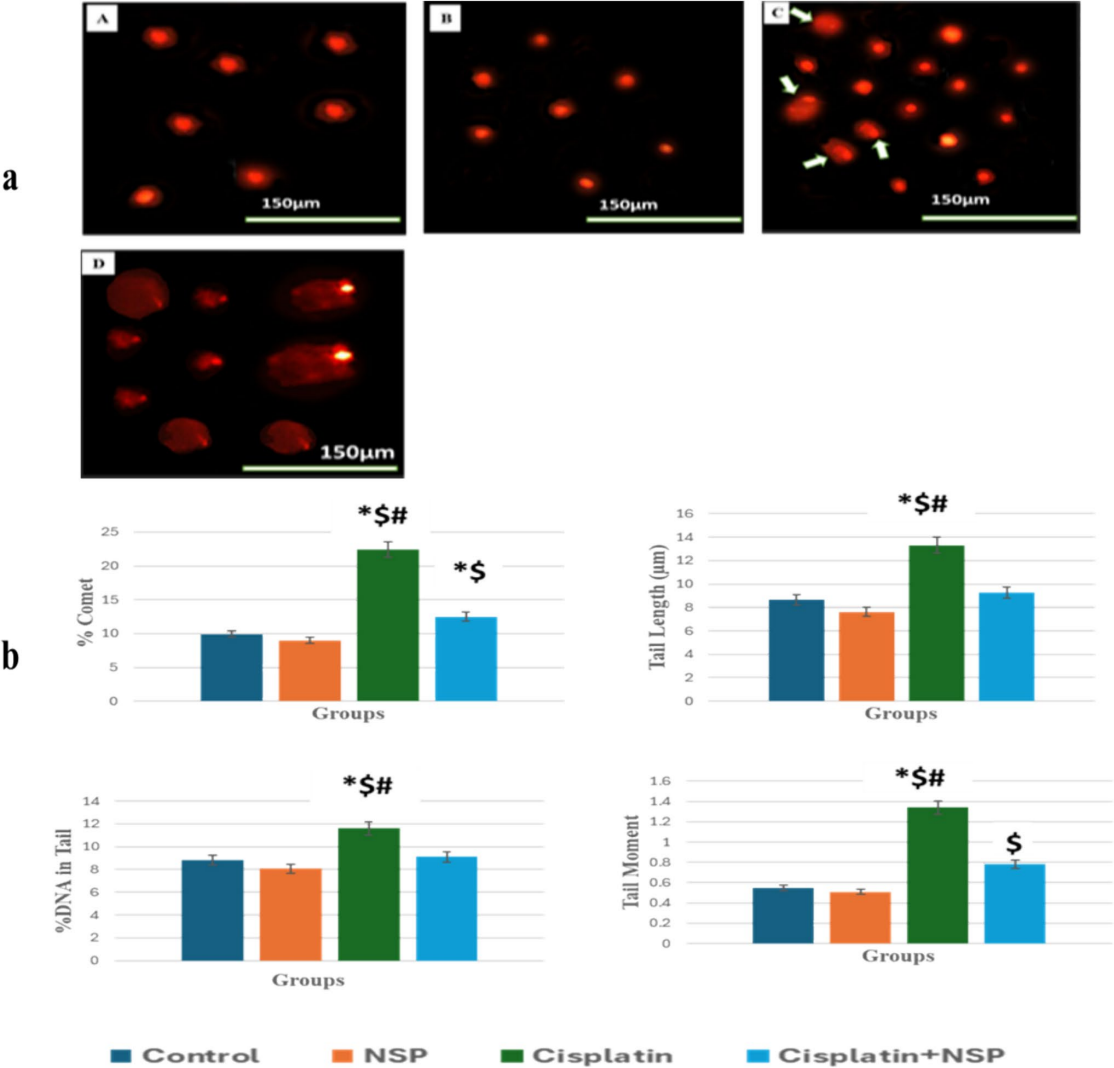
(**a**) Representative photos of DNA damage in liver tissue of 20-day-old fetuses induced by cisplatin (D: cisplatin group, compared with undamaged DNA in control group (A) and NSP group (B)). C: cisplatin + NSP group showed decreased DNA damage (arrows). (**b**) DNA damage represented as % comet, tail length, %DNA in tail, and tail moment in the rat testicular tissues of different groups. Results are expressed as mean ± standard error of mean, * means statistically significant as compared to control; $ means statistically significant as compared to the NSP group; # means statistically significant as compared to the cisplatin + NSP group at *p* < 0.05

Testicular tissue from cisplatin-treated rats showed considerable DNA damage as represented by comet percentage, tail length, and percentage of DNA in the tail and tail moment compared to both control and NSP groups (Fig. 6).

#### StAR and SOD gene expression

**Fig. 7:**
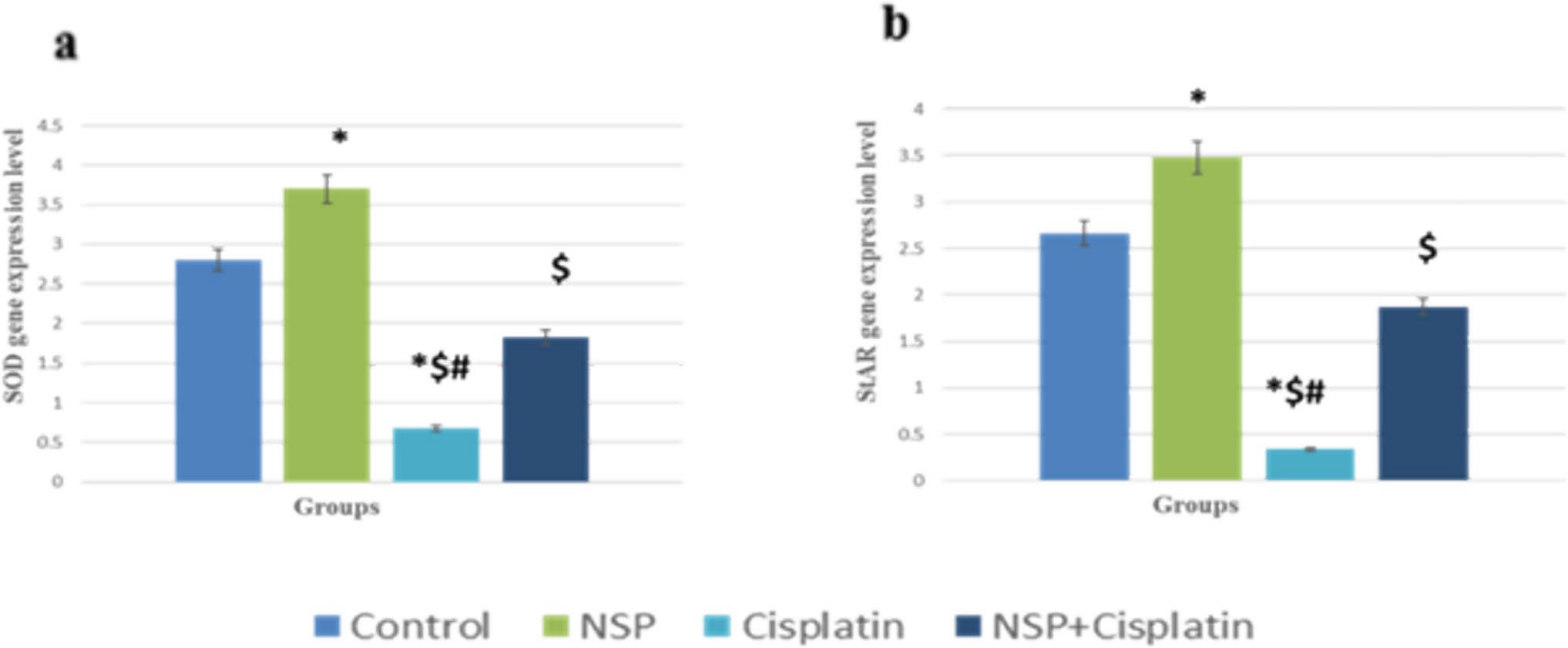
Effect of cisplatin on the expression of **a** StAR and **b** SOD. Results are expressed as mean ± standard error of mean, * means statistically significant as compared to control; $ means statistically significant as compared to the NSP group; # means statistically significant as compared to the cisplatin + NSP group at *p* < 0.05

The result indicated that cisplatin considerably decreased the mRNA concentration level of both StAR and SOD genes. However, the expression of StAR and SOD genes were returned after NSP intake compared to the cisplatin-treated animals (Fig. 7).

## Discussion

Recent work has determined if NSP leaf extract could potentially protect male albino rats’ testicular tissue from the dangerous effects of cisplatin. Smaller materials and nanomaterials scale between 1 and 1000 nm have a larger surface area to volume ratio. This enhances conduction and bio-dispersion throughout organs and tissues and the elimination rate (Gera et al. 2017). As a result, the NSP employed in this work might exhibit higher bioactivity due to its minute particles (68 nm).

According to the World Health Organization (WHO), cancer is the primary reason of death worldwide, with about 10 million dead people by 2020 (WHO 2022). According to Dasari and Tchounwou (2014), cisplatin is among the most effective anticancer drugs versus solid cancers in both children and adults. It is frequently linked to several toxic side effects, including gonadal toxicity (Okada and Fujisawa 2019).

In the present experiment, rats treated with cisplatin had significantly lower body weights and absolute reproductive organ weights. Some researchers thought body weight loss was a confidential sign of toxicity (Garcia et al. 2013; Ito et al. 2020). The protective impact of NSP on body weight may be attributed to the body’s strengthening with key nutritional compounds. It has essential compounds, such as vital amino acids (Farag et al. 2016), nutraceutical pigments (Keservani et al. 2015), essential fatty acids, gamma-linolenic and linoleic, alpha-linolenic (Mendes et al. 2003), vitamins such as vitamins A, D E, thiamine, riboflavin, nicotinamide, pyridoxine folic acid (Hosseini et al. 2013), and minerals like Fe, P, Na, Mg, Zn, Se, Cu, Ca, K, Cr, and Mn (Babadzhanov et al. 2004).

Cisplatin injection in the present work led to a substantial decrease in both the testis weight and epididymal sperm quality and deleterious effects in testicular tissues when compared to the control group. These results were consistent with Mesbahzadeh et al. (2021). After entering the cell, cisplatin’s chloride groups are replaced by water molecules; these hydrophilic modifications facilitate the incorporation of this potent chemotherapeutic medication into cellular macromolecules and nitrogen donor atoms on nucleic acids (Kartalou 2001). The latter would hinder spermatogenesis and development of spermatogonia stem cells and Leydig cells resulting in azo- or oligospermia (Cherry et al. 2004; Howell and Shalet 2005) and decreased testosterone synthesis (Ito et al. 2020) by ROS-mediated P450scc suppression in testicular Leydig cells (Garcia et al. 2013). Cisplatin causes a significant increase in sperm abnormalities due to its excessive production of ROS (Dasari and Tchounwou 2014), which can lead to oxidative stress (Sies 2015). In coincidence with the present study, Aitken (2017) stated that oxidative stress causes injury to spermatozoa by elevating lipid peroxidation levels and changing sperm membrane function with disturbed morphology, metabolic activity, motility, and fertilization capacity.

On the other hand, NSP has two antioxidant defense systems, enzymatic (ascorbate peroxidase, CAT, SOD, and GSH) and non-enzymatic bioactive materials such as β-carotene, chlorophylls, and C-phycocyanin (Hlima et al. 2019) in addition to anti-lipid peroxidation functions (Kurd and Samavati 2015; El-Tantawy 2016). The pigment materials in NSP scavenge a diversity of reactive species and enhance endogenous enzymatic antioxidants (Gabr et al. 2020). Adding *Spirulina platensis* extract to the diet enhances boar semen quality (Kistanova et al 2009) and rabbits (El-Ratel and Gabr 2020) and improves rat spermatogenesis (Esener et al. 2017).

Glutathione peroxidase (GSHPX) functions on the decrease of both hydrogen peroxide and organic peroxides (Walczak-Jedrzejowska et al. 2013). It has three isoforms: nuclear, cytosolic, and mitochondrial. The mitochondrial isoform is needed for sperm motility and quality (Rehman et al. 2020). High NSP zinc levels play a key role in the spermatozoa’s physiological functions, as well as the stabilization of sperm membranes (Chia et al. 2000). Sperms obtain energy from fructose sugar produced by the seminal vesicles and ductus deferens ampulla for metabolic processes and spermatozoa movement (Schoenfeld et al. 1979) and might be a useful indication of a healthy male reproductive state (Toragall et al. 2019). In our experiment, there was a significant increase in fructose concentration in the cisplatin group, which was represented by the oligospermia group, compared to the untreated group. This agreed with Trang et al. (2018), who observed that seminal fructose concentration has adverse relationships with sperm motility and concentration. In the cisplatin + NSP group, the concentration of fructose became significantly lower than in the cisplatin group as NSP restored sperm motility and concentration.

The histopathological results revealed amorphous shapes of seminiferous tubules with exfoliated germ cells within the luminal region, decreased interstitial cells, and a marked disturbance in the spermatogenic cell pattern, proving the decline in sperm quality indicators in rats given cisplatin alone. Moreover, cisplatin boosted testicular destruction, germinal epithelium height shrinking, and spermatogenesis impairments that were authorized by a significant drop in Johnsen’s score (Lopes et al. 2021b). The significant correlation between testicular damage and reduced Johnsen score of spermatogenesis was cited in several studies (Mesbahzadeh et al. 2021; Ismail et al. 2023; Nofal et al. 2023). Decreasing germinal epithelium height in cisplatin-injected animals is respected as the final step of the degeneration process caused by cisplatin (Okada and Fujisawa 2019). The germinal epithelial cells were exfoliated into the lumen of the seminiferous tubules due to apoptosis and degeneration of these cells due to cisplatin action (Saral et al. 2016; Meligy et al. 2019; Nofal et al. 2023). Moreover, cisplatin disrupts the mitochondria respiratory chain, resulting in apoptosis and oxidative stress in testis tissue (Ismail et al. 2023).

However, NSP restored the testicular architecture due to its bioactive components. For example, phenols in *Spirulina* acted as ROS scavengers (Roy et al. 2015); vitamin E (α-tocopherol) and ascorbic acid increased testicular antioxidant properties against lead-induced testicular damage in rats (Ayinde et al. 2012). The high concentration of NSP zinc improves the structural integrity of germinal epithelium and keeps the reproductive organs lined (Fallah et al. 2018).

The testicular tissues of the cisplatin group revealed a statistically significant increase in comet percent, tail length, percentage of DNA in the tail, and tail moment. Previous findings have revealed that cisplatin has a property of direct DNA damage and connects not only with the nuclear DNA but also to the mitochondrial DNA (Cullen et al. 2007; Marullo et al. 2013; Wang et al. 2018). This results in the production of DNA adducts, which inhibit the cell cycle and lead to cytotoxicity in Sertoli cells, testicular germinal cells, and Leydig cells, ending in azoospermia and/or oligospermia and testicular injuries (Howell and Shalet 2005; Meistrich 2013; Harman and Richburg 2014).

Experiments have proved that NSP antioxidant supplements can inhibit or lessen DNA damage (Niki 2010). *Spirulina* has vitamins A, C, D, B1, B2, B3, B6, B9, and E (Chamorro et al. 1996; Babadzhanov et al. 2004). Vitamin E in *Spirulina* could also prevent DNA damage and its lethal effects on the body. *Spirulina* can directly interact with free radicals, altering lipid peroxides to hydroxyl resin (Habib et al. 2008).

The current investigation found a noticeable decline in TS and DHEA levels in rats injected with cisplatin, hence impeding the maturation of sperm and triggering physiological abnormalities in the sex organs. Testosterone increases protein production in all spermatogonia and significantly functions in sperm production (Ismail et al. 2023). As a result, a drop in testosterone inhibits protein formation in germinal cells, resulting in adverse outcomes, as reported in rodents (Saral et al. 2016). Androstenedione is a steroid hormone produced by the adrenal glands and gonads of both males and females (Malaviya and Gomes 2008). 17-Beta hydroxysteroid dehydrogenase acts as a catalytic agent in transforming androstenedione to testosterone using DHEA (Luu-The 2001) (Fig. 4). Any functional disturbances in Leydig cells are responsible for decreased serum testosterone concentrations because most of androgen manufacture and release in males occurs in them (Baulieu et al. 2000; Liu et al. 2015).

Cisplatin may suppress androgen receptor mRNA expression in Sertoli cells, which affects TS (Ganong 2011). Furthermore, cisplatin could reduce the concentration of TS by dropping the ratio of testicular zinc, which inhibits the converting enzyme of angiotensin (Huang et al. 2008). The significant decrease in DHEA level in the cisplatin group may be due to disturbances in the enzymatic functions of 3ß-HSD, 17ß-HSD, and aromatase, which are present in Leydig cells and catalyze the formation of testosterone and estradiol (Liu et al. 2015; Kumar et al. 2008). Abnormal levels of 17β-HSD 13 in the cisplatin group may be responsible for lower testosterone production. NSP treatment restored the TS, DHEA, and 17β-HSD 13 concentrations.

Both hydroxysteroid dehydrogenase (HSD) and cytochrome P450 (CYP) are the major enzymes in steroid biosynthesis. Pregnenolone represents a precursor for the entire steroidal hormones. 17α-Hydroxylase converts pregnenolone into 17α-hydroxy pregnenolone. 3β-HSD can oxidize pregnenolone and synthesize progesterone, converts into deoxycorticosterone, and gives DHEA and androstenedione (Chakraborty et al. 2021). 17-Beta hydroxysteroid dehydrogenase (17β-HSD) catalyzes the conversion of androstenedione to testosterone using DHEA (Luu-The 2001).

In the current work, cisplatin increased MDA levels while dramatically decreasing antioxidants including CAT, GSH, and SOD. Elevated ROS levels can damage mitochondrial DNA (Bui et al. 2018), influencing sperm physiological function (Wang et al. 2022). Catalase is one of the fundamental antioxidant enzymes that moderates oxidative stress to a significant production of water and oxygen from destroying hydrogen peroxide in the cells (Nandi et al. 2019). The primary role of GSH is its antioxidant competence, which helps sustain the cellular redox cycle (Georgiou-Siafis and Tsiftsoglou 2023). SOD plays a guiding role in the shielding of plasma membrane constituents, DNA breakage, and polyunsaturated fatty acids (Rehman et al. 2020).

Cisplatin administration produced excessive ROS, reduced antioxidant activity, and increased lipid peroxidation in rat testes (Kohsaka et al. 2020). Reactive oxygen species and OS cause drug cytotoxicity, inducing injury to bioactive molecules and disruption to the oxidant/antioxidant equilibrium (Hashem et al. 2019). On the other hand, NSP restored the concentration of these antioxidants as it contains several enzymatic antioxidants, including GSH, ascorbate peroxidase, CAT, and SOD, and it has an anti-lipid peroxidation function that reduced the MDA level (Kurd and Samavati 2015; El-Tantawy 2016).

In recent work, reducing the expression of the StAR gene and disturbance in the StAR protein concentrations in the testicular tissue resulted in a significant reduction in testosterone levels because expression of StAR is primarily associated with steroid production (Jana et al. 2010; Zhao et al. 2013). Steroidogenesis begins with cholesterol transferring to the inner mitochondrial membrane by StAR, afterward transformed to pregnenolone by P450 (Miller and Bose 2011; Manna et al. 2013; Jana et al. 2014). The current investigation found that cisplatin treatment reduced the gene expression of the StAR proteins, as mentioned by Yilmaz et al. (2018). Reduced StAR gene expression was associated with testicular damage (Luo et al. 2011; Ebiya et al. 2016). However, NSP raised the expression of the StAR gene when matched to the cisplatin-treated rats, as stated by Nah et al. (2012). *Spirulina platensis* contains flavonoids (Rahim et al. 2021) and quercetin (Wang 2011; Martin and Touaibia 2020; Rashwan and Hammad 2020) that promote StAR transcription by preventing Dax-1 expression (Martin and Touaibia 2020) and increase Creb1 transcriptional activity, supporting steroidogenesis (Cormier et al. 2018).

Superoxide dismutase enzymes are plentiful in sperm cells and semen plasma; SOD in semen is associated with sperm count and mobility in a positive manner (Buffone et al. 2012; Lopes et al. 2021a). If this antioxidant enzyme undergoes polymorphisms, this will lead to infertility in human males (Lopes et al. 2021a; Bach et al. 2023). The result of our study revealed that cisplatin caused a significant decrease in SOD gene expression. However, this reduction was restored after NSP administration in the combined group, as NSP contains SOD (Hlima et al. 2019). Thus, an improvement in sperm concentration and motility was observed in the cisplatin + NSP group.

## Conclusion

The existing study examined the ameliorating effect of NSP against testicular damage, spermatogenesis malfunction, and decreased fertility resulting from cisplatin administration. The cisplatin-treated group showed numerous histological, histometric, and biochemical irregularities and DNA damage. Administration of NSP improved spermatogenesis, overcame testicular impairment, decreased oxidative stress, raised testosterone hormone concentration in blood, restored the concentration of 17-ß-HSD, promoted germinal cells, upgraded sperm quality, boosted antioxidant enzymatic activity, and upregulated the SOD and StAR genes. These results suggested that NSP is a hopeful and appurtenant antioxidant against male infertility. Thus, due to its unique antioxidant properties, NSP may benefit chemotherapeutic drugs associated with infertility.

## Data Availability

The datasets used and/or analyzed during the current study are available from the corresponding author upon reasonable request.
